# Psilocin acutely alters sleep-wake architecture and cortical brain activity in laboratory mice

**DOI:** 10.1038/s41398-022-01846-9

**Published:** 2022-02-23

**Authors:** Christopher W. Thomas, Cristina Blanco-Duque, Benjamin J. Bréant, Guy M. Goodwin, Trevor Sharp, David M. Bannerman, Vladyslav V. Vyazovskiy

**Affiliations:** 1 Department of Physiology, Anatomy and Genetics, University of Oxford, Oxford, UK; 2 Department of Psychiatry, University of Oxford, Oxford, UK; 3 Department of Pharmacology, University of Oxford, Oxford, UK; 4 Department of Experimental Psychology, University of Oxford, Oxford, UK

**Keywords:** Pharmacology, Molecular neuroscience

## Abstract

Serotonergic psychedelic drugs, such as psilocin (4-hydroxy-N,N-dimethyltryptamine), profoundly alter the quality of consciousness through mechanisms which are incompletely understood. Growing evidence suggests that a single psychedelic experience can positively impact long-term psychological well-being, with relevance for the treatment of psychiatric disorders, including depression. A prominent factor associated with psychiatric disorders is disturbed sleep, and the sleep-wake cycle is implicated in the homeostatic regulation of neuronal activity and synaptic plasticity. However, it remains largely unknown to what extent psychedelic agents directly affect sleep, in terms of both acute arousal and homeostatic sleep regulation. Here, chronic electrophysiological recordings were obtained in mice to track sleep-wake architecture and cortical activity after psilocin injection. Administration of psilocin led to delayed REM sleep onset and reduced NREM sleep maintenance for up to approximately 3 h after dosing, and the acute EEG response was associated primarily with an enhanced oscillation around 4 Hz. No long-term changes in sleep-wake quantity were found. When combined with sleep deprivation, psilocin did not alter the dynamics of homeostatic sleep rebound during the subsequent recovery period, as reflected in both sleep amount and EEG slow-wave activity. However, psilocin decreased the recovery rate of sleep slow-wave activity following sleep deprivation in the local field potentials of electrodes targeting the medial prefrontal and surrounding cortex. It is concluded that psilocin affects both global vigilance state control and local sleep homeostasis, an effect which may be relevant for its antidepressant efficacy.

## Introduction

Psilocybin is a classical serotonergic psychedelic. A growing body of evidence suggests that, under appropriate conditions, psilocybin exposure promotes long-lasting positive effects on psychological well-being, offering a promising new treatment method for affective disorders [1–4]. Induction of the psychedelic state depends on the metabolite of psilocybin, psilocin (4-hydroxy-N,N-dimethyltryptamine), which acts as a partial agonist of 5-HT_2A_ receptors [5–7]. Psychedelics act on 5-HT_2A_ receptors on layer V pyramidal neurones, inhibitory interneurones, and presynaptic thalamocortical afferents across the cortex, most notably prefrontal cortex [8–11], modulating glutamate transmission [12–14] and disrupting neural dynamics and functional networks on a brain-wide scale [15–23]. However, the challenge remains to dissect which neuronal effects are specific to, and necessarily characteristic of, the psychedelic state [24, 25]. The psychedelic state is widely suggested to promote neuroplasticity, which is theorised to be important for psilocybin’s therapeutic efficacy [26]. While the evidence demonstrates that psychedelics can induce structural and functional synaptic plasticity in vivo [27–29], facilitate learning and memory [30–32], and exert long-lasting behavioural effects [33, 34] in rodents, the specific underlying neurophysiology remains unclear, especially regarding the relevance to psychiatric disorders in humans.

Neuronal plasticity is not passive but is shaped by ongoing neuronal activity. The brain-wide changes in neuronal activity occurring alongside the sleep-wake cycle are strongly implicated in the regulation of plasticity of cortical function; for example, cellular maintenance, synaptic scaling, firing rate homeostasis, and systems-level memory consolidation are enabled by the sleep state [35–40]. Alongside the circadian rhythm, the occurrence of sleep is itself regulated by a homeostatic principle; prolonged wakefulness is compensated by increased sleep intensity, enabling an approximately constant sleep quantity to be obtained each day [41]. The brain’s homeostatic sleep debt is reflected during non-rapid eye movement (NREM) sleep in the average levels of slow-wave activity (0.5-4 Hz) in neurophysiological field potentials [42–44], such as the electroencephalogram (EEG) or intracortical local field potential (LFP). However, slow-wave amplitude and dynamics across the cortical surface reveal a heterogeneity and dependence on both behaviour and neuronal activity levels during previous wakefulness, with evidence for a bidirectional relationship between local neuronal activity and sleep-wake homeostasis [45–49].

Sleep disturbance is strongly associated with the development and maintenance of many common psychological disorders, including depression [50–53]. The depressed state has been theorised to be characterised by an impairment in sleep homeostasis, for example that the need for sleep increases more slowly during wakefulness and so is chronically low in depressed patients [54–56]. It is unclear whether sleep changes in depression represent disease symptoms or adaptive mechanisms which develop to counteract depressive pathophysiology. The latter possibility may explain why, somewhat paradoxically, acute sleep deprivation exerts a rapid anti-depressive effect which typically relapses after subsequent sleep occurs [56].

Currently little is known about the effects of psychedelics on sleep regulation or the degree to which their enduring psychological benefits are sleep-dependent [57]. Psilocybin does acutely affect sleep in humans [58], however, animal models will be necessary to understand the underlying mechanisms, which may yield insights into the core plasticity processes involved in the aetiology of, and recovery from, disordered brain states such as depression. A few early studies report acute EEG changes and sleep disruption in animals in response to psychedelic 5-HT_2A_ receptor agonists [59–61], however, the wider implications for homeostatic sleep regulation and possible enduring effects remain to be explored. Here, we characterise acute and enduring changes to sleep-wake-related behaviour and electrophysiology in mice following injection of psilocin, in both an undisturbed and a sleep-deprived condition. We found that psilocin acutely disrupted sleep maintenance and promoted quiet wakefulness. This state was associated with altered power spectra in frontal and occipital EEG derivations and in LFPs targeted in and around the medial prefrontal cortex, notably including an enhanced 3–5 Hz rhythm and reduction in gamma-band power. Despite the acute sleep disturbance, psilocin administration was not associated with long-term changes to sleep-wake architecture. After 4 h of sleep deprivation paired with psilocin exposure, no difference was observed in slow-wave activity at the EEG level. However, after psilocin injection, a reduced rate of recovery of slow-wave activity during sleep was found in the LFP, implicating intriguing way in which the sleep homeostatic process might be altered by psychedelic compounds.

## Methods

### Surgical Procedures

Eight young adult male C57BL/6 J mice (aged 14–20 weeks) were surgically implanted with electrodes for the continuous recording of electroencephalography (EEG) and electromyography (EMG), as well as with either a microwire array (*n* = 4) or single-shank electrode (*n* = 4) targeting the medial prefrontal cortex.

All procedures were performed under a UK Home Office Project License and conformed to the Animals (Scientific Procedures) Act 1986. Surgeries were performed under isoflurane anaesthesia (4% induction, 1–2% maintenance). Analgesics were administered immediately before surgery (5 mg/kg metacam and 0.1 mg/kg vetergesic, subcutaneous) and for at least three days following surgery (metacam, oral). In addition, an immunosuppressant was given both the day before surgery (0.2 mg/kg dexamethasone, intraperitoneal) and immediately before surgery (0.2 mg/kg dexamethasone, subcutaneous).

EEG screw electrodes were implanted above the right frontal cortex (primary motor area: anteroposterior 2 mm, mediolateral 2 mm), right occipital cortex (primary visual area: anteroposterior 3.5 mm, mediolateral 2.5 mm), left cerebellum (reference signal), and left occipital cortex (ground for intracortical electrodes). EMG wires were inserted into the left and right nuchal muscle. Four animals were implanted with a single-shank probe in left anterior medial cortex, targeting cingulate, prelimbic and infralimbic cortex (anteroposterior 1.7 mm, mediolateral 0.25 mm, depth 2 mm). The probe comprised a 5 mm shank containing 16 iridium electrode sites of 30 μm diameter, regularly spaced 50 μm apart and extending up to 800 μm from the probe’s tip (A1x16-5mm-50–703, NeuroNexus, Michigan, USA). The remaining four animals were implanted with a custom-designed polyimide-insulated tungsten microwire array (Tucker-Davis Technologies Inc., Florida, USA), spanning a larger area of left anterior medial cortex (centered anteroposterior 2.23 mm, mediolateral 0.75 mm, depth 2.2 mm, rotation 10 degrees). The array comprised 16 wire channels of 33 μm diameter arranged in 2 rows of 8, with row separation 375 μm, columnar separation 250 μm, and tip angle 45 degrees. Each wire was a custom-specified length for precise targeting of prefrontal regions (lateral row from anterior to posterior: 3.2 mm, 3.5 mm, 3.5 mm, 3.8 mm, 3.8 mm, 4 mm, 4 mm, 4 mm; medial row from anterior to posterior: 2.5 mm, 2.8 mm, 3 mm, 3.2 mm, 3.2 mm, 3.5 mm, 3.5 mm, 3.5 mm). For the single-shank probe, an additional hole was drilled to the size of the probe. For the arrays, a 1 × 2.25 mm craniotomy window was drilled into the skull. Once the array/probe was implanted, a silicone gel (KwikSil, World Precision Instruments, Florida, USA) was applied to seal the craniotomy and protect the exposed brain. Dental acrylic was used to stabilise the implanted electrodes (Super Bond, Prestige Dental, Bradford, UK) and to protect the exposed wires (Simplex Rapid, Kemdent, Swindon, UK).

### Animal Husbandry

Following surgery, mice were housed separately in individually ventilated cages and their well-being was monitored until recovery remained at baseline level for three consecutive days. Animals were then rehoused in individual plexiglass cages (20.3 × 32 × 35 cm), placed inside ventilated sound-attenuated Faraday chambers (Campden Instruments, Loughborough, UK). Mice were cabled and habituated for three days before the first baseline recording began. The recording room was kept on a 12–12 h light-dark cycle (lights on at 9 am), at 22 ± 1 ^o^C and 50 ± 20 % humidity. Food and water were provided *ad libitum* throughout. Video of the animals was recorded continuously during the light period.

### Experimental Design

Animals underwent four injection experiments, comprising two sleep-wake conditions and two drug treatments, using a within-subjects design. In one sleep-wake condition, mice were immediately returned to their home cage after injection and left undisturbed. In the second condition, a sleep deprivation protocol was enforced for 4 h after injection, using a well-established procedure of gentle handling and novel object presentation [47]. Each injection experiment occurred over 3 days, following a pattern of baseline day, injection day, and recovery day. There were no off days, meaning that 72 h passed between injections and all four experiments were concluded within 12 days. All injections were delivered immediately after light onset. The experiments were run with two cohorts of four animals. The same sleep-wake condition was always run back to back so that no animal received two psilocin injections sequentially. Sleep-wake condition order was reversed across the two cohorts and drug treatment order was counterbalanced across the animals (Table 1). Animals were assigned to their ordering of experimental conditions following the order in which surgery was performed, which was pseudorandom (i.e. animal 1 in Table 1 was the first animal on which surgery was performed, animal 2 the second, and so on). Experimenters were not blinded to treatment.

**Table 1:** Counterbalancing of treatment order.

Animal	First Experiment	Second Experiment	Third Experiment	Fourth Experiment
**1**	A	B	C	D
**2**	B	A	D	C
**3**	A	B	C	D
**4**	B	A	D	C
**5**	D	C	B	A
**6**	C	D	A	B
**7**	C	D	A	B
**8**	D	C	B	A

A = Undisturbed + Vehicle; B = Undisturbed + Psilocin; C = Sleep deprivation + Vehicle; D = Sleep Deprivation + Psilocin.

### Preparation of Drugs

Psilocin (4-hydroxy-N,N-dimethyltryptamine) solution was prepared immediately before each injection experiment. Crystalline psilocin (LGC Standards) was dissolved in 50 mM tartaric acid and subsequently diluted in saline (5% glucose) up to a final psilocin concentration of 0.25 mg/ml. The vehicle solution was also prepared fresh before each experiment by diluting an equal volume of 50 mM tartaric acid in saline (final tartaric acid concentration at injection was 0.625 mM in both solutions). All drugs were administered by intraperitoneal injection as this is most practical for tethered animals. Psilocin was administered at a dose of 2 mg/kg.

### Data Acquisition

Electrophysiological signals were acquired using a multichannel neurophysiology recording system (Tucker-Davis Technologies Inc., Florida, USA). Signals were managed and processed online using the software package Synapse (Tucker-Davis Technologies Inc., Florida, USA). All signals were amplified (PZ5 NeuroDigitizer preamplifier, Tucker-Davis Technologies Inc., Florida, USA), filtered online (0.1–128 Hz) and stored with a sampling rate of 305 Hz. Raw LFP signal was additionally processed to detect extracellular multi-unit spiking activity, by filtering (300 Hz–1 kHz) and manual amplitude thresholding. Whenever threshold crossing occurred the time stamp and signal snippets (46 samples at 25 kHz, 0.48 ms before and 1.36 ms after threshold crossing) were stored.

Signals were read into Matlab and filtered with a zero-phase 4th order Butterworth filter (0.5–100 Hz for EEG/LFP, 10–45 Hz for EMG), then resampled at 256 Hz. Spiking activity from each channel was cleaned offline for artefacts using the Matlab spike sorting software *Wave_clus* [62], although quality single units were not found. Multi-unit firing rate was calculated in epochs of 4 s separately for each channel. Spike rates were normalised as a percentage of the channel mean within the same vigilance state at baseline prior to averaging across channels.

### Sleep Scoring

**Fig. 1:**
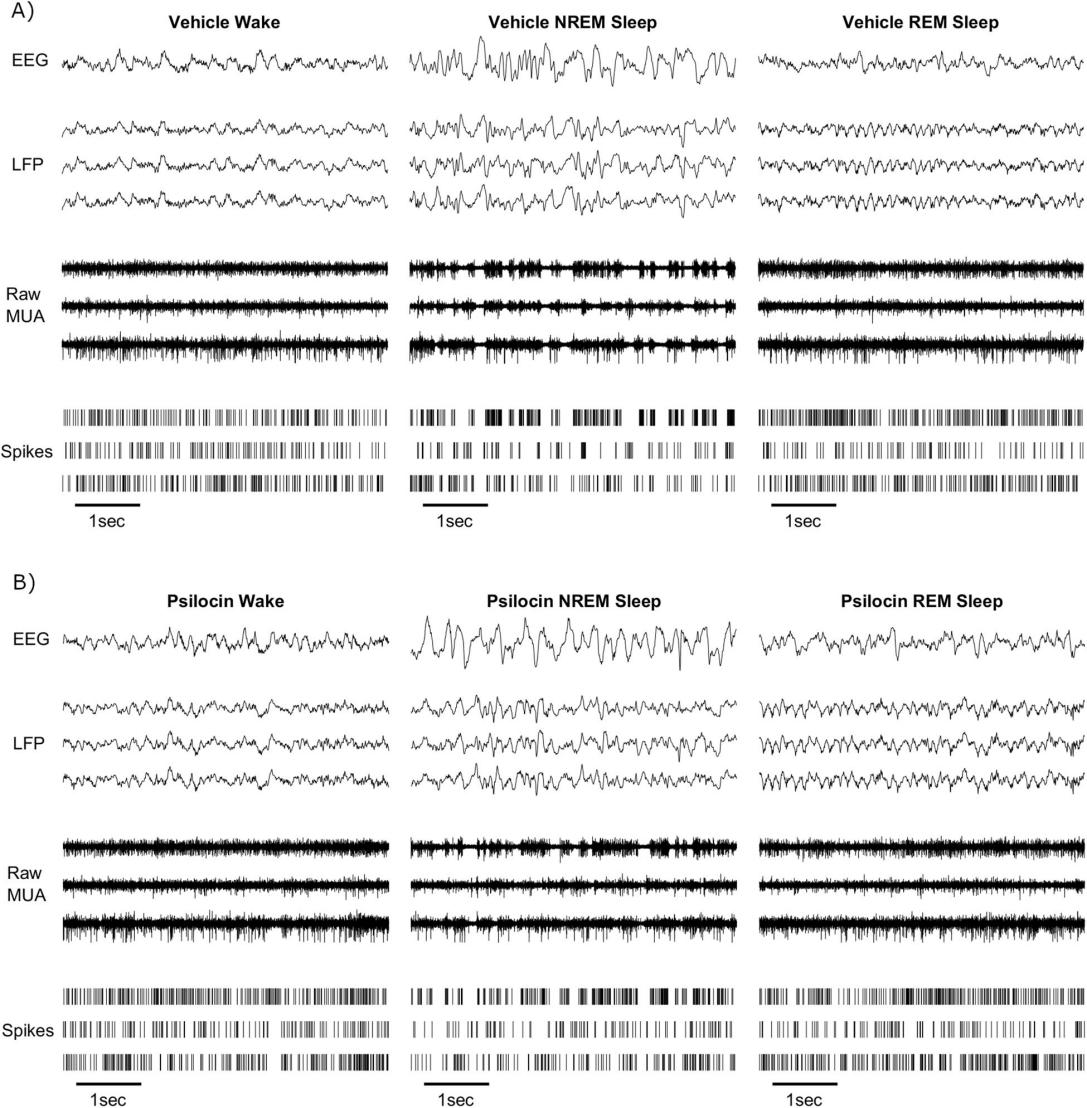
Example wake, NREM and REM sleep signals following vehicle and psilocin injection. An example segment of 5 s duration of frontal electroencephalogram (EEG), 3 cortical local field potentials (LFP), corresponding raw signal with multi-unit activity (MUA) and detected spikes in representative segments of waking, NREM and REM sleep, approximately 10, 40, and 70 min respectively, after **A**) injection with vehicle solution, **B**) injection with psilocin.

Vigilance states were scored manually by visual inspection at a resolution of 4 s, following conventional practice, using the software SleepSign (Kissei Comtec, Nagano, Japan). Wake was characterised by low amplitude irregular EEG and LFP signals alongside asynchronous high-frequency multi-unit spiking. In contrast, NREM sleep was identifiable by the presence of high amplitude EEG and LFP slow waves coincident with synchronous spiking multi-unit off periods. REM sleep periods were identifiable by a reduced slow-wave activity, increased theta power, and readily distinguishable from waking by low EMG levels and sleep-wake context (Fig. 1). To exclude time periods with large amplitude artefacts across LFP channels, a hybrid LFP signal was created comprising the maximum absolute value of any one LFP at each time point and plotted alongside EEG for manual artefact scoring. For most analyses of NREM sleep, as indicated, continuous bouts of NREM sleep less than 1 min in duration were excluded, following convention, as full state transition unfolds over this approximate time course [63].

### Histology

After completing recording, the animals were euthanised, transcardially perfused with paraformaldehyde (PFA, 4%) and their brains were prepared for histological analysis for the location of inserted probes. Extracted brains were sectioned into 50 µm coronal slices using a freezing microtome and stained with DAPI. Probes were stained before insertion with DiI. The slices were imaged using an Olympus FV1000 confocal microscope and compared with an anatomical atlas [64] to aid localisation of probes. Histology was successful in four animals (two single shank and two array). Based on this subset, the estimated distribution of targeted cortical regions was approximately prelimbic (39%), cingulate (31%), secondary motor (12%), medial orbital (10%), and infralimbic (8%). Histological analysis of the remaining animals suggested that some electrodes may have reached deeper and more posterior structures, including the dorsal striatum and lateral septal nucleus, however, this could not be definitively confirmed. For analysis, all quality LFP signals were grouped and treated as one population. Our aim was not to investigate any particular brain region in and around prefrontal cortex, or make comparisons between regions, but to contrast locally derived signals with those from the frontal and occipital EEG.

### Data Inclusion/Exclusion

Out of the 8 animals in the study, frontal EEG recordings were successfully obtained from 7, occipital EEG from 5, LFP from 6, and multi-unit spiking activity from 7. Lost signals were due to damage to the electrode or connecting wires. All signals were obtained simultaneously from 5 animals. All individual LFP signals were manually examined and a total of 15 (out of 112) were identified for exclusion based on the presence of frequent high amplitude artifacts or unsystematic drift in signal amplitude during key analysis time windows.

### Field Potential Spectral Analysis

EEG and LFP spectra were analysed with a discrete fast Fourier transform (FFT) on segments of 4-second duration, applying a Hann window. Spectral power values were averaged over epochs scored as the same vigilance state within the time window of interest separately for each animal. An average power for each discrete frequency value in wake, NREM and REM sleep was calculated for each animal from the whole 24-h baseline day before psilocin injection. For plotting spectra, values between 45 and 55 Hz were interpolated for ease of visualisation, since power in this frequency range was removed by a notch filter targeting 50 Hz line noise. Slow-wave activity was obtained for each epoch by summing power over frequencies from 0.5–4 Hz.

### Statistics

Statistical tests were all performed using Matlab functions (*anova2* for 2-way ANOVA, *ttest* for paired samples *t*-tests and *ranksum* for Wilcoxon tests). For ANOVA, the associated F-statistic is reported, which is a ratio of between-group variance and within-group variance. Both the between- and within-group degrees of freedom are given in subscript. In some cases, where indicated, a log transform was applied to improve data normality. Lilliefors test was used to determine data normality and the nonparametric Wilcoxon test applied instead of a *t*-test when significant data non-normality was found. When testing for differences between power spectra t-tests were run at all individual discrete frequency values, applying a *p* < 0.05 significance threshold. No correction for multiple comparisons was applied, following convention, as this is typically too conservative when spectra are compared in this way owing to the number of frequency bins. All tests were two-sided. The sample size was chosen to reflect the typical number of animals used in exploratory in vivo rodent electrophysiology studies, power calculations were not performed.

## Results

### Sleep is acutely destabilised and fragmented by psilocin

In the first experimental condition, mice were injected with psilocin (2 mg/kg, i.p.) or vehicle at the start of the light phase (when the animals typically sleep) and immediately left undisturbed in their home cage. The animals were necessarily awakened by the injection and psilocin’s most striking behavioural effect was to disrupt the first attempts at reinitiating sleep. Psilocin-affected mice spent a significant amount of time in their nests, adopting a posture compatible with sleep, but still apparently awake according to electrophysiological criteria. Rest in these animals was frequently disturbed by small body movements, such as stretches and readjustments of posture, and often the eyes remained open even while motionless (Supplementary Video 1). Among the motor disturbances was a sporadic head twitch response, as has been previously characterised [65, 66], with more frequent head twitches counted in the psilocin condition across the 30 minute period immediately following injection (Vehicle: 0.34 ± 0.27 min^−1^; Psilocin: 0.73 ± 0.13 min^−1^; p = 0.014, paired *t*-test).

**Fig. 2:**
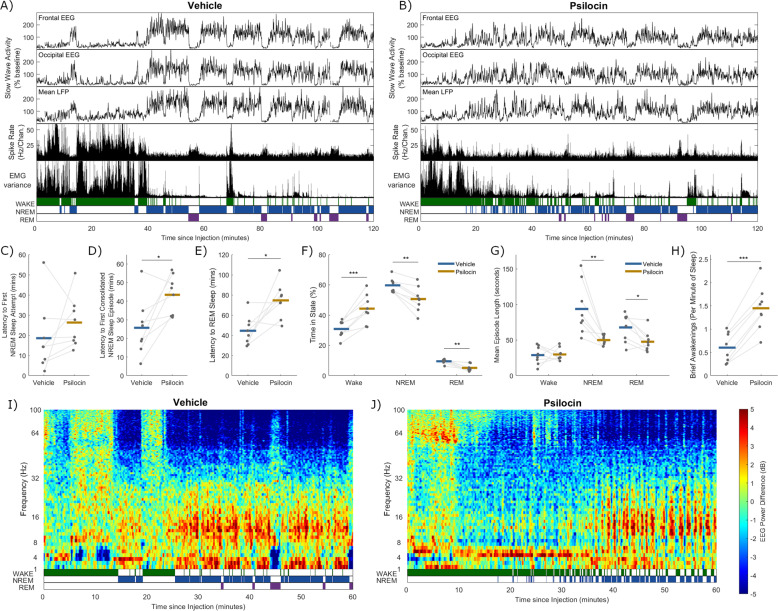
Acute disruption of sleep-wake behaviour and brain activity by psilocin. A representative example of slow-wave activity (0.5–4 Hz power) derived from frontal electroencephalogram (EEG), occipital EEG, and mean local field potential (LFP), alongside the total recorded spike firing rate (spikes per second per channel), variance of the electromyogram (EMG) and scored vigilance states, all with a resolution of 4 seconds over a period of 2 h after **A**) injection with vehicle, and **B**) injection with psilocin, in the undisturbed condition. The latency in minutes from injection **C**) until the first 4-second epoch scored as NREM sleep, **D**) until the first continuous NREM sleep episode at least 1-minute duration, and **E**) until the first 4-second epoch scored as REM sleep. **F**) The percentage of the three-hour period after injection which was scored as wake, NREM or REM sleep, and **G**) the mean length in seconds of wake, NREM and REM sleep episodes in this time. **H**) The number of brief awakenings (wake episodes < 20 seconds occurring within NREM episodes at least 1-minute duration) per minute of NREM sleep during the first hour after injection. In **C-H**) grey dots correspond to individual animals, with grey lines linking values from the same animal, coloured lines indicate the group mean for vehicle (blue) and psilocin (yellow) conditions and asterisks denote statistical significance with a paired t-test; *: *p* < 0.05, **: *p* < 0.01, ***: *p* < 0.001. Time-frequency plots characterising changes in frontal EEG spectral power (in decibels relative to power averaged over the baseline day) over the first hour after injection with **I**) vehicle and **J**) psilocin, from one representative example (different animal to **A** & **B**).

Psilocin did not significantly change the essential electrophysiological features of wake, NREM and REM sleep (Fig. 1), and it was therefore possible to score sleep-wake episodes. Analysis of acute sleep-wake activity using electrophysiological criteria suggested that the psilocin-injected animals were rapidly alternating between short wake and shallow NREM sleep episodes (Fig. 2A, B). The average latency from injection to the first 4-s NREM sleep epoch was not significantly different between conditions (Vehicle: 18.6 ± 6.1 min, Psilocin: 26.3 ± 4.5 min, *p* = 0.30, *n* = 8, paired *t* test, Fig. 2C). However, latency from injection to the first continuous NREM sleep episode of at least 1-min duration, was significantly increased after psilocin (Vehicle: 25.7 ± 5.4 min, Psilocin: 43.4 ± 3.7 min, *p* = 0.015, *n* = 8, paired *t-*test, Fig. 2D). Similarly, the latency to the initiation of any REM sleep was also increased by psilocin (Vehicle: 44.5 ± 5.1 min, Psilocin: 74.6 ± 6.1 min, *p* = 0.013, *n* = 8, paired *t*-test, Fig. 2E).

Alterations to sleep-wake activity in the psilocin-treated animals were observed to be greatest during the first hour after injection but could last up to approximately 3 h, and so this time window was analysed further. Over the 3 h following injection, psilocin increased the average proportion of time spent awake (Vehicle: 30.8 ± 2.0%, Psilocin: 44.3 ± 3.4%, *p* = 6.8 ×10^−4^, *n* = 8, paired *t*-test), and correspondingly significantly decreased the time spent in NREM (Vehicle: 59.7 ± 1.7%, Psilocin: 50.6 ± 2.9%, *p* = 0.0011, *n* = 8, paired *t*-test), and REM sleep (Vehicle: 9.4 ± 0.5%, Psilocin: 5.1 ± 0.8%, *p* = 0.0012, *n* = 8, paired *t*-test, Fig. 2F). During this period, the mean duration of continuous wake episodes was unchanged (Vehicle: 28.8 ± 4.5 s, Psilocin: 29.8 ± 3.1 s, *p* = 0.89, paired *t-*test), whereas episode duration was significantly reduced for both NREM (Vehicle: 93.6 ± 12.9 s, Psilocin: 50.0 ± 2.6 s, *p* = 0.0070 paired *t*-test) and REM sleep (Vehicle: 67.7 ± 7.0 s, Psilocin: 47.6 ± 5.1 s, *p* = 0.019, Fig. 2G). This form of sleep disruption resembles an increased propensity for brief awakenings, usually defined in mice as periods of wakefulness lasting ≤ 20 seconds occurring during NREM sleep and typically accompanied by small body movements. During the first hour following injection, the frequency of brief awakenings in NREM sleep was increased by psilocin (Vehicle: 0.60 ± 0.32 min^−1^; Psilocin: 1.4 ± 0.48 min^−1^; *p* = 2.0 ×10^−5^, *n* = 8, paired *t-*test, Fig. 2H). These results suggest that the increased wakefulness produced by psilocin is due to an increased drive to awaken from sleep, corresponding to an impairment of sleep maintenance rather than an enhanced stability of wakefulness.

This period of rapidly alternating wake and NREM sleep is further illustrated in Fig. 2I, J, showing a time-frequency plot for the frontal EEG spectral power (relative to the baseline day), from one representative example animal after both vehicle and psilocin administration. In the vehicle condition, clear vigilance state boundaries are visible in the spectrogram (Fig. 2I), including wake periods with heterogenous spectral composition, NREM sleep with increased low frequency (<30 Hz) and decreased high-frequency power (>30 Hz) and REM sleep characterised by reduced low frequency (<6 Hz) and elevated upper theta (7–10 Hz) power. In contrast, in the psilocin condition, the wake to sleep transition was less distinct (Fig. 2J). In this example, psilocin injection is followed by approximately 10 min of active wakefulness characterised by elevated theta (5–9 Hz) and upper gamma (> 50 Hz) power. Subsequently, a quiet wake period occurs containing frequent NREM sleep attempts but dominated by wakefulness, generally characterised by reduced low-frequency power (<30 Hz) and elevated power in a narrow band around approximately 4 Hz. Approximately 35 min post-injection, consolidated NREM sleep becomes more distinct, indicated by increased low frequency (<30 Hz) and decreased high-frequency power (>30 Hz), although frequent brief awakenings persist. No REM sleep occurred in this example.

### Long-term sleep-wake architecture is unaffected by psilocin

**Fig. 3:**
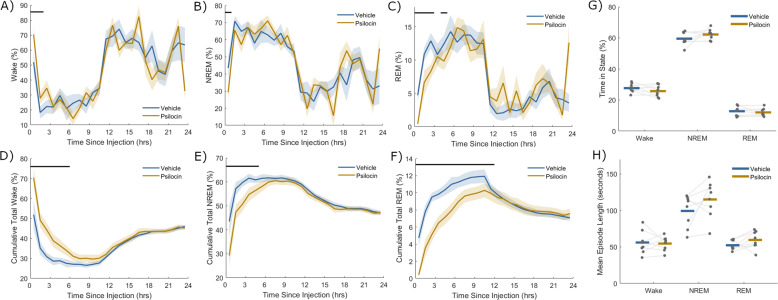
Sleep-wake architecture over 24 hours following vehicle and psilocin injection. Percentage of time scored as **A**) wake, **B**) NREM sleep and **C**) REM sleep in successive non-overlapping windows of one-hour up to 24 h after injection with vehicle (blue) and psilocin (yellow). The total cumulative percentage of time scored as **D**) wake, **E**) NREM sleep and **F**) REM sleep from injection until up to 24 h after injection with vehicle (blue) and with psilocin (yellow), as a function of time since injection. Note that at each time point cumulative total of wake, cumulative total of NREM sleep and cumulative total of REM sleep will together always sum to 100%. Coloured lines denote the mean and sections the standard error of the mean over all animals. Black lines indicate time points that were significantly different (*p* < 0.05) according to paired *t*-tests applied at discrete time points. **G**) The percentage time from three hours after injection until the end of the light period which was scored as wake, NREM or REM sleep, and **H**) the mean length in seconds of wake, NREM and REM sleep episodes in this time. Grey dots correspond to individual animals, with grey lines linking values from the same animal, coloured lines indicate the group mean for vehicle (blue) and psilocin (yellow) conditions.

The hour-by-hour distribution of wake, NREM and REM sleep averaged over animals for 24 h after injection is shown in Fig. 3A–C. A clear light-dark cycle is evident, in which animals are awake more throughout the dark phase, particularly during its first half (beginning between 11 and 12 h after injection). Overall, there is no striking change in sleep-wake architecture due to psilocin on this time scale, except for the increase in wake and suppression of sleep, particularly REM sleep, in the first few hours. Importantly, there is no specific time point following the acute disruption of sleep at which a rebound in NREM or REM sleep is evident. To visualise the restoration of vigilance state homeostasis, the percentage of time since injection in each state was plotted as a function of time since injection over 24 h. These cumulative time courses of wake, NREM and REM sleep (Fig. 3D–F) suggest that homeostasis of vigilance state quantity is restored within one day.

From three hours after injection until the end of the light period, the total fraction of time spent in each vigilance state was not significantly different between psilocin and vehicle conditions (Wake: *p* = 0.25, NREM: *p* = 0.24, REM: *p* = 0.42, *n* = 8, paired *t*-test, Fig. 3G). Additionally, the average duration of wake, NREM and REM sleep episodes was unchanged (Wake: *p* = 0.79, NREM: *p* = 0.13, REM: *p* = 0.13, *n* = 8, paired *t*-test) (Fig. 3H). Similarly, the quantity of wake, NREM and REM sleep was not different in the dark period after injection (Wake: *p* = 0.50, NREM: *p* = 0.92, REM: *p* = 0.07, *n* = 4, paired *t-*test).

### Psilocin differentially affects the sleep homeostatic process manifesting at different spatial scales between EEG and LFP

The sleep-wake history of an individual is tracked by physiological processes in the brain in order to homeostatically regulate global vigilance states, such that, for example, sleep deprivation is compensated by increased subsequent sleep duration and intensity. This phenomenon is termed “Process S”, and with an underlying biological substrate that is not completely certain, measures the magnitude of the homeostatic drive to sleep, and can predict with high accuracy EEG slow-wave activity through mathematical models [42–44, 48, 63].

Given the pronounced acute effects of psilocin on sleep-wake states observed in the first experiment, we hypothesised that the sleep homeostatic process (Process S) would also be affected. To explore this and address the confound of psilocin’s acute direct effects on arousal, in the second experimental condition, mice were injected as before at light onset with either 2 mg/kg psilocin or vehicle, and immediately kept awake for 4 h by engaging the animals with presentation of novel objects. We aimed to determine whether sleep quantity or slow-wave activity levels would differ in subsequent recovery sleep between drug and vehicle conditions.

**Fig. 4:**
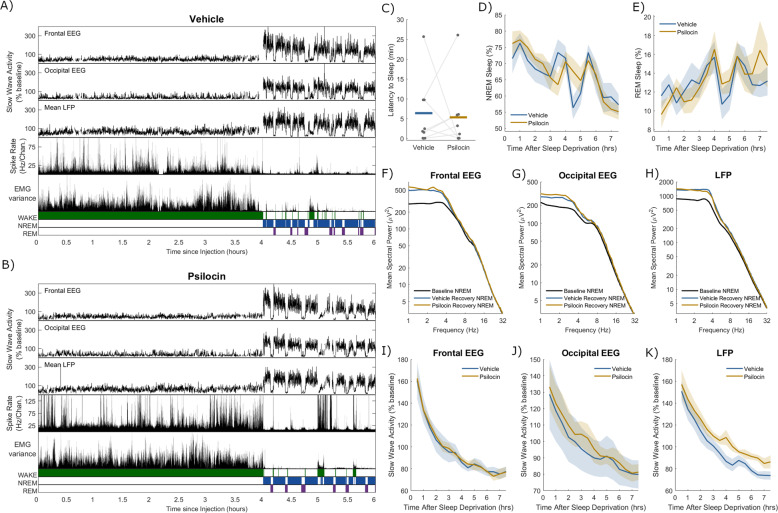
Recovery sleep after sleep deprivation paired with vehicle and psilocin injection. A representative example of slow-wave activity (0.5–4 Hz power) derived from frontal electroencephalogram (EEG), occipital EEG, and mean local field potential (LFP), alongside the total recorded spike firing rate (spikes per second per channel), the variance of the electromyogram (EMG) and scored vigilance states, all with a resolution of 4 s over a period of 6 h comprising 4 h of sleep deprivation and 2 h of recovery sleep, including **A**) injection with vehicle, and **B**) injection with psilocin. **C**) Latency from the end of sleep deprivation to the first episode of NREM sleep at least 1-minute duration. Grey dots correspond to individual animals, with grey lines linking values from the same animal, coloured lines indicate the group mean for vehicle (blue) and psilocin (yellow) conditions. The percentage of time scored as **D**) NREM sleep and **E**) REM sleep from the end of sleep deprivation until the end of the light period. Coloured lines denote the mean and ranges the standard error of the mean. The mean power spectra of **F**) frontal EEG, **G**) occipital EEG and **H**) mean LFP in NREM sleep in the first period of sleep after the end of sleep deprivation, compared with mean spectra over all NREM sleep the baseline day. The time series of slow-wave activity (0.5–4 Hz power, relative to that on the baseline day) derived from **I**) frontal EEG, **J**) occipital EEG and **K**) mean LFP, from the end of sleep deprivation until the end of the light period. Individual time points correspond to averages of slow-wave activity in overlapping 1-hour windows centred every 30 min.

Overall, the electrophysiological signals during recovery sleep were similar between vehicle and psilocin conditions and the expected increased slow-wave activity indicating elevated Process S was consistently observed during NREM sleep after sleep deprivation (Fig. 4A, B). After 4 h of sleep deprivation the median latency to the initiation of NREM sleep was not significantly different between psilocin and vehicle groups (Vehicle: 2.1 min, Psilocin: 2.2 min, *p* = 0.84, *n* = 8, Wilcoxon signed rank test, Fig. 4C). Sleep quantities were analysed during the remainder of the light period in 30 min time bins to identify whether differences in sleep might manifest at specific time points. There was an effect of time, reflecting the expected early dominance of NREM sleep followed by increasing prevalence of REM sleep, but not of drug condition or their interaction for both NREM (Drug: *F*_(1,210)_ = 0.28, *p* = 0.60; Time: *F*_(14,210)_ = 6.11, *p* < 0.001; Interaction: *F*_(14,210)_ = 0.89, *p* = 0.57; two-way ANOVA, Fig. 4D) and REM sleep (Drug: *F*_(1,210)_ = 0.15, *p* = 0.70; Time: *F*_(14,210)_ = 2.16, *p* = 0.011; Interaction: F_(14,210)_ = 0.6, *p* = 0.86; two-way ANOVA, Fig. 4E).

While this result suggests that Process S was unaffected by psilocin administration, changes may still be visible at the level of localised cortical activity. Power spectra were calculated by averaging over NREM sleep in the first recovery sleep episode (end of sleep deprivation to first wake episode at least 5 min duration, Vehicle: 1.47 ± 0.7 h, Psilocin: 1.24 ± 0.4 h). The expected elevations in slow-wave activity relative to baseline were seen in frontal EEG, occipital EEG and mean LFP, but no significant differences were observed between psilocin and vehicle conditions (Fig. 4F, G, H). Furthermore, the time course of average slow-wave activity in NREM sleep over the remainder of the light period after sleep deprivation further shows no effect of psilocin, only of time, in both frontal EEG (Fig. 4I; Drug: *F*_(1,120)_ = 0.09, *p* = 0.76; Time: *F*_(14,120)_ = 27.3, *p* < 0.001; Interaction: *F*_(14,120)_ = 0.05, *p* = 1; two-way ANOVA) and occipital EEG (Fig. 4J; Drug: *F*_(1,90)_ = 0.97, *p* = 0.33; Time: F_(14,90)_ = 3.7, *p* < 0.001; Interaction: *F*_(14,90)_ = 0.04, *p* = 1; two-way ANOVA). However, a significant effect for psilocin was found in the time course of mean LFP slow-wave activity (Drug: *F*_(1,150)_ = 23.0*, p* < 0.001; Time: *F*_(14,150)_ = 24.0, *p* < 0.001; Interaction: *F*_(14,150)_ = 0.19, *p* = 0.99; two-way ANOVA), which exhibited a reduced rate of decrease in the psilocin condition (Fig. 4K). To confirm this result we calculated the rate of decline of mean LFP SWA in recovery after sleep deprivation by fitting an exponential curve to the data from each individual animal, finding significantly lower exponential time constants in the psilocin condition (vehicle: mean = 0.11 h^−1^, psilocin: mean = 0.09 h^−1^, *p* = 0.028, paired *t*-test). To explore whether this effect is present in the prefrontal cortex, this analysis was repeated including only animals with confirmed electrode placements in prefrontal (prelimbic and infralimbic) cortex, finding the same significant effect of psilocin and a decreased decay rate of SWA during recovery sleep after sleep deprivation (Drug: *F*_(1,90)_ = 16.7, *p* < 0.001; Time: F_(14,90)_ = 17.6, *p* < 0.001; Interaction: F_(14,90)_ = 0.14, *p* = 0.99; two-way ANOVA). This result implies that Process S recovered more slowly during sleep after sleep deprivation combined with psilocin, but only on a local level, at least in prefrontal and adjacent cortex, and not at the global level as measured with EEG.

### Electrophysiological characteristics of the psilocin-induced state

**Fig. 5:**
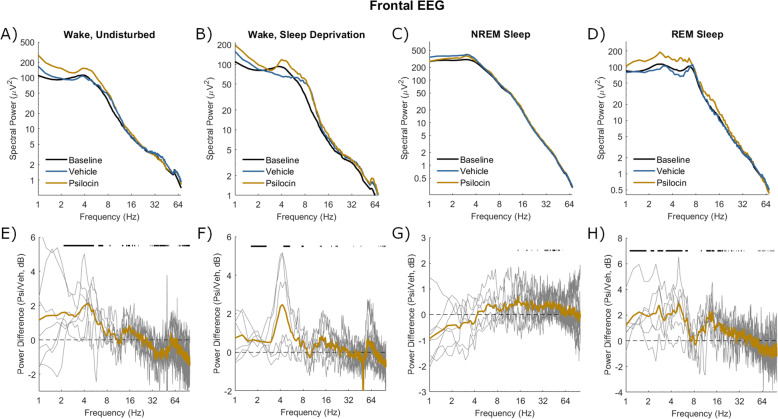
Spectral power in frontal EEG after vehicle and psilocin injection. The mean power spectra of frontal EEG in four different vigilance state conditions following injection with vehicle (blue) and psilocin (yellow). **A**) ‘Wake, undisturbed’ corresponds to the first experiment, all wake epochs from injection until the first NREM episode at least 1-minute duration. **B**) ‘Wake, sleep deprivation’ corresponds to the first 30 min of sleep deprivation in the second experiment. **C**) ‘NREM sleep’ and **D**) ‘REM sleep’ correspond to the first experiment, all NREM/REM sleep epochs in the sleep period after injection, defined from the start of the first NREM sleep episode at least 1 min duration until the next wake episode at least 5 min duration. Spectra averaged across the same vigilance state in the baseline day are shown for comparison (black). **E**–**H**) Below each plot illustrates the spectral power difference as a function of frequency in decibels between vehicle and psilocin conditions (positive is greater after psilocin). Grey lines correspond to individual animals and coloured lines to the mean. Black lines indicate discrete frequencies (at 0.25 Hz resolution) that were significantly different (*p* < 0.05) according to paired *t*-tests.

**Fig. 6:**
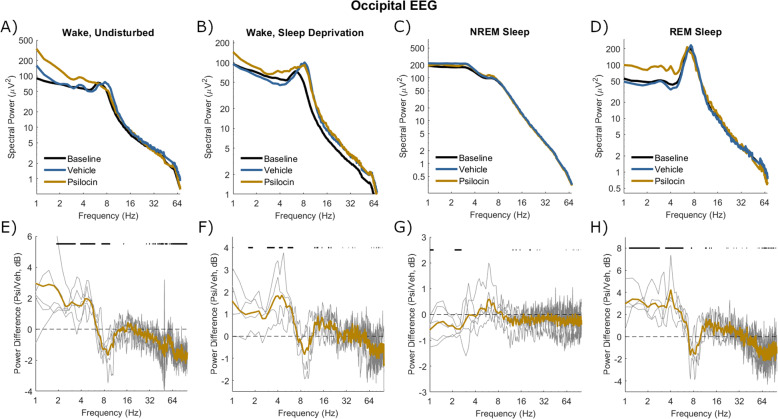
Spectral power in occipital EEG after vehicle and psilocin injection. The mean power spectra of occipital EEG in four different vigilance state conditions following injection with vehicle (blue) and psilocin (yellow). **A**) ‘Wake, undisturbed’ corresponds to the first experiment, all wake epochs from injection until the first NREM episode at least 1-minute duration. **B**) ‘Wake, sleep deprivation’ corresponds to the first 30 min of sleep deprivation in the second experiment. **C**) ‘NREM sleep’ and **D**) ‘REM sleep’ correspond to the first experiment, all NREM/REM sleep epochs in the sleep period after injection, defined from the start of the first NREM sleep episode at least 1 min duration until the next wake episode at least 5 min duration. Spectra averaged across the same vigilance state in the baseline day are shown for comparison (black). **E**–**H**) Below each plot illustrates the spectral power difference as a function of frequency in decibels between vehicle and psilocin conditions (positive is greater after psilocin). Grey lines correspond to individual animals and coloured lines to the mean. Black lines indicate discrete frequencies (at 0.25 Hz resolution) that were significantly different (*p* < 0.05) according to paired *t*-tests.

**Fig. 7:**
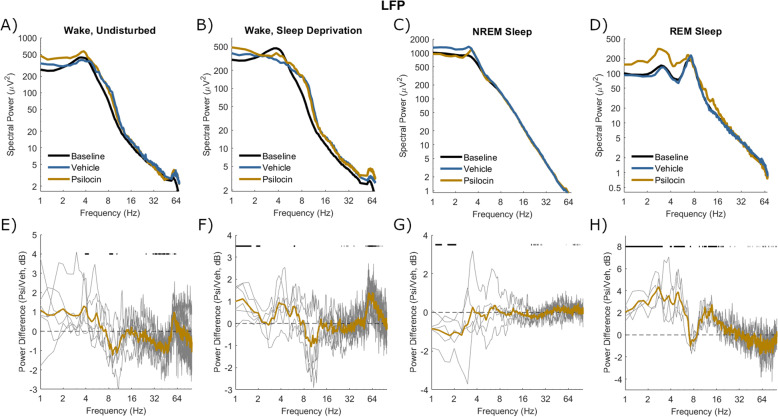
Spectral power in the mean LFP after vehicle and psilocin injection. The mean power spectra of mean LFP in four different vigilance state conditions following injection with vehicle (blue) and psilocin (yellow). **A**) ‘Wake, undisturbed’ corresponds to the first experiment, all wake epochs from injection until the first NREM episode at least 1-minute duration. **B**) ‘Wake, sleep deprivation’ corresponds to the first 30 min of sleep deprivation in the second experiment. **C**) ‘NREM sleep’ and **D**) ‘REM sleep’ correspond to the first experiment, all NREM/REM sleep epochs in the sleep period after injection, defined from the start of the first NREM sleep episode at least 1 min duration until the next wake episode at least 5 min duration. Spectra averaged across the same vigilance state in the baseline day are shown for comparison (black). **E**–**H**) Below each plot illustrates the spectral power difference as a function of frequency in decibels between vehicle and psilocin conditions (positive is greater after psilocin). Grey lines correspond to individual animals and coloured lines to the mean. Black lines indicate discrete frequencies (at 0.25 Hz resolution) that were significantly different (*p* < 0.05) according to paired *t*-tests.

The effects of psilocin on EEG and LFP spectra in different states of vigilance were then explored. Wake was analysed in both the undisturbed condition (from injection until the first NREM sleep attempt, Vehicle: 18.6 ± 17.2 min, Psilocin: 26.3 ± 12.7 min) and sleep deprivation condition (first 30 min after injection). In both wake conditions, baseline spectra from frontal EEG and LFP were characterised by a peak around 4 Hz (Figs. 5A, B, 7A, B). Enhancement of power around 3–5 Hz by psilocin was evidenced in the waking frontal EEG, and in the undisturbed condition in the LFP (Figs. 5A, B, 7B). Notably this peak was reduced in the sleep deprivation condition in the LFP and with the vehicle in frontal EEG (Figs. 5B, 7B). Low-frequency power increases in occipital EEG were broader and at a high frequency with psilocin (Fig. 6A, B), reflecting widening of the theta (5–8 Hz) peak present in baseline spectra. These changes are likely linked to behaviour, for example, the 3–5 Hz rhythm may be associated with quiet wakefulness [67, 68], and indeed a negative correlation was found between 3-5 Hz frontal EEG power and EMG variance per 4-s epoch as a measure of motor activity during the 3-hour period after psilocin injection (Spearman’s *R* = −0.47 ± 0.12, *n* = 5). High-frequency power in the gamma range (>30 Hz) was generally decreased by psilocin in both EEG derivations and to a greater degree in the undisturbed condition (Figs. 5A, B, 6A, B). This effect was weaker in the LFP and accompanied by an increase in high-frequency power (>60 Hz) particularly during the sleep deprivation condition (Fig. 7B).

Both NREM and REM sleep were analysed in the undisturbed condition from the first episode of NREM sleep after injection of at least 1-minute duration, to the next wake episode at least 5 min duration (NREM Vehicle: 66.9 ± 22.2 min; NREM Psilocin: 52.2 ± 20.8 min; REM Vehicle: 10.1 ± 5.5 min, REM Psilocin: 7.3 ± 4.8 min). In NREM sleep, no well-defined band-specific differences were identified between vehicle and psilocin conditions. A trend existed in all EEG and LFP signals for decreased low frequencies (<4 Hz), perhaps reflecting that sleep was less intense (Figs. 5C, 6C, 7C). During REM sleep after psilocin, high frequencies (>30 Hz) tended to be reduced, whereas low frequencies (< 8 Hz and 10–20 Hz) were mostly increased (Figs. 5D, 6D, 7D). These differences might be interpreted as a bleeding of NREM-like activities (delta waves, spindles, reduced gamma) into REM sleep.

### Dynamics of the 3-5 Hz oscillation across sleep deprivation

**Fig. 8:**
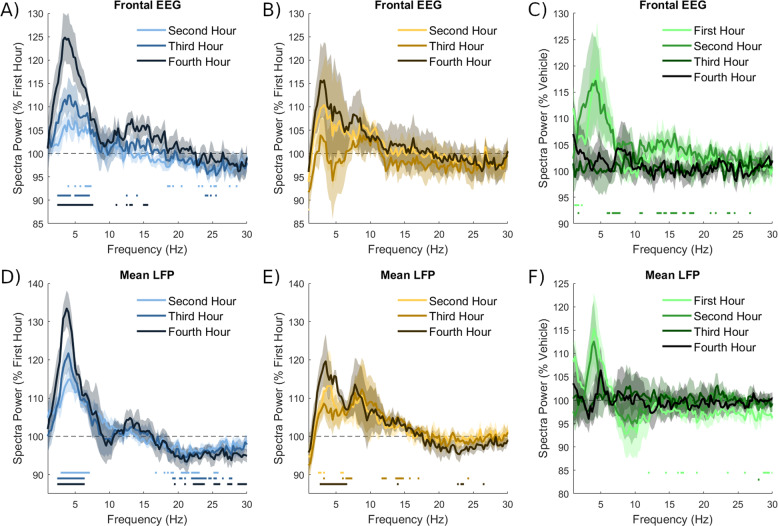
The 3-5 Hz oscillation across sleep deprivation following vehicle and psilocin injection. The spectral power as a function of frequency across the 4 h of sleep deprivation, **A**) in the frontal EEG in the vehicle and **B**) psilocin condition. Values plotted are averages in the second, third and fourth hours as a percentage of average values in the first hour. **C**) Values plotted are averages in the four individual hours in the psilocin condition as a percentage of those in the corresponding hour in the vehicle condition. **D, E, F**) show the same calculated for the mean LFP. Bold lines represent the mean and coloured ranges the standard error of the mean. Lines underneath the plots indicate discrete frequencies (at 0.25 Hz resolution) that were significantly different (*p* < 0.05) from 100% according to a *t*-test.

An increase in oscillatory power around 4 Hz, as was found to be elicited by psilocin, has previously been associated with drowsiness, the accumulation of sleep pressure and occurrence of local sleep [67]. To investigate whether this oscillation could be related to the sleep homeostatic process we compared its dynamics over the sleep deprivation period in both drug conditions. Spectra were averaged within one-hour windows and expressed as a percentage of power in the first hour, or hour by hour as a percentage of power in the vehicle condition at the equivalent time. As expected, in the vehicle condition, a low-frequency band centred around 4 Hz increased in power hour by hour during sleep deprivation in both the frontal EEG (Fig. 8A) and mean LFP (Fig. 8D). However, this pattern was not observed in the psilocin condition, owing to the elevation of power in this frequency band in the first hour from the acute drug effects. There is a trend for elevation of power in this band in the frontal EEG (Fig. 8B), and it is significantly higher in the fourth sleep deprivation hour in the LFP (Fig. 8E). These dynamics likely result from the summation of two opposing processes, which regulate oscillations in this 3–5 Hz frequency range; the drop in power over time owing to metabolic clearance of psilocin is approximately balanced by the continuing build-up of sleep pressure. In the final 2 h of sleep deprivation, spectra were not different between vehicle and psilocin conditions in frontal EEG (Fig. 8C) or LFP (Fig. 8F), suggesting that once the acute drug effects wear off, similar levels of Process S remain in both conditions. The results suggest that this low-frequency oscillation is induced directly by psilocin over the first 2 h and by mounting sleep pressure in the final 2 h, with a net effect of little hour by hour change.

## Discussion

### Effects of serotonergic agents and antidepressants on sleep

This work has explored the effects of psilocin, a psychedelic 5-HT_2A_ receptor agonist, on sleep-wake regulation, sleep homeostasis and associated cortical activity in mice. The role of the serotonin system in sleep-wake control is complex and somewhat controversial [69, 70]. Serotonergic raphe neurones are more active during wakefulness than sleep and serotonin is widely included in the monoaminergic ascending arousal system thought to maintain wakefulness [71]. An acute sleep-suppressing effect of psychedelic 5-HT_2A_ receptor agonists has been previously reported in rodents and cats [59–61]. Correspondingly, 5-HT_2A_ receptor antagonists are sleep-promoting in both rats and mice [61, 72], while in humans, 5-HT_2A_ receptor antagonists increase the depth and maintenance of sleep and have been explored in the treatment of insomnia [73, 74]. In this study, serotonergic stimulation with psilocin injection produced an expected increase in wakefulness, more accurately characterised as sleep fragmentation through disruption of NREM and REM sleep maintenance.

REM sleep suppression is a common side-effect of many classical antidepressants, such as selective serotonin reuptake inhibitors, which are slow-acting and dosed chronically [75, 76], a somewhat paradoxical phenomenon given the positive association between REM sleep and emotional regulation [77]. Disruption of REM sleep after psychedelics has been reported in humans, albeit only in the night after drug exposure [58, 78]. Similarly, REM sleep suppression in this study lasted only on the order of hours. Given the limited duration of effects, it is unlikely that modulation of REM sleep quantity is a core mechanism of the psychological benefits of psychedelics, although it remains possible that the underlying brain activity of REM sleep is affected in a more subtle way.

### Does psilocin affect Process S?

While serotonin agonists are acutely wake promoting, early raphe lesion studies implicated a role in sleep promotion as well [69, 70]. Recent optogenetic experiments in mice have discriminated wake-promoting burst activity in the raphe, from tonic activity which increases sleep drive [79]. Indeed, the 5-HT_2A_ receptor may be involved, as 5-HT_2A_ receptor knockout mice sleep less and exhibit an attenuated homeostatic sleep rebound [72]. Furthermore, psychedelics promote plasticity, and regulation of synaptic strengths is one possible substrate of Process S [37, 80]. Although the relationship between plasticity and sleep regulation is neither straightforward nor fully understood [81], it can be hypothesised that psilocin would disturb sleep homeostasis.

Analysis of frontal EEG and LFP spectra in these recordings identified a prominent 3–5 Hz peak, present at baseline but amplified by psilocin. As it resembles the oscillation known to reflect local sleep and the build-up of sleep pressure [67], it is possible that it represents an adaptive process by which sleep pressure is able to be dissipated in parallel with its build-up. However, there exist alternative interpretations of such an oscillation, for example, activity in this frequency range has been previously associated with breathing during wakefulness in mice in the prefrontal cortex [82], as well as other areas [83, 84] and serotonergic signalling is implicated in breathing regulation [85]. The prefrontal respiratory rhythm emerges during wake immobility, synchronises with nasal breathing and modulates ongoing prefrontal cortical gamma activity and spike timing [82]. This respiratory rhythm has also been linked with neocortical-hippocampal communication, plasticity, and memory [86, 87]. A recent study reported periods of EEG oscillatory activity at around 4 Hz in mice after treatment with a 5-HT_2A_ receptor agonist, finding that these coincide with behavioural inactivity, although breathing was not measured [68]. That this psilocin-associated oscillation might be related to local sleep or respiration remains to be directly tested, and its functional significance within the context of both endogenous 5-HT_2A_ receptor activation and the psychedelic phenomenon is unclear. Dissecting the behavioural and pharmacological influences on this oscillation would require more carefully controlled experiments.

Evidence for an interaction between psychedelics and sleep homeostasis can be found in previous human studies. One reported elevated EEG slow-wave activity during sleep 11 h after ingestion of ayahuasca, a traditional psychedelic drink containing dimethyltryptamine [78]. However, another human study with psilocybin reported that EEG slow-wave activity was actually suppressed in the first cycle of NREM sleep [58]. Here, in mice, Process S measured in this typical way at the “global level” (with EEG slow-wave activity) was not impacted by psilocin, and similarly no enduring effects of psilocin were observed on sleep-wake architecture in either experimental condition. These differences may be due to species or drug dose, or even the duration between injection and sleep; shorter durations run the risk that acute drug effects confound arousal whereas longer durations may produce a ceiling effect on slow-wave amplitude reducing signal to noise ratio for effect detection. It is also likely that the effects of psychedelics on arousal and sleep regulation might depend on circadian time and preceding sleep-wake history since, for example, 5-HT_2A_ receptor binding increases after sleep deprivation [88]. Careful control of these factors will be essential in future studies.

It is further possible that these inconsistent results might reflect the coarse spatial scale over which Process S is typically considered. In this regard, the finding of a reduced recovery rate of Process S in the LFP is of potential importance. The global Process S manifesting in EEG slow-wave activity may result from the integration across the brain of many local Processes S, which in turn each reflect the recent history of local neuronal activities [48]. If the rate of Process S recovery is slowed locally in prefrontal regions, it is possible that recovery may occur more quickly elsewhere in the brain, such as posterior cortex. This is a testable hypothesis and an in-depth mapping of Process S across the cortical surface would be necessary before inference can be made into the functional significance in the context of regional specialisation. Importantly, since slow-wave activity indicates elevated neuronal synchronisation, local variation in Process S may well be linked to neuroplasticity and the finding here is consistent with the widely held view that the prefrontal cortex is a key cortical region affected by psilocybin.

### Future outlook

Psychedelic drugs such as psilocin provide a novel approach to study the basic science underpinning sleep regulation, offering a means to manipulate the content of wakefulness and associated brain dynamics. Psychedelic stimulation offers important advantages compared to other manipulations of waking brain activity, such as optogenetic activation of cortical neurones, owing to its relative simplicity, physiological validity, translatability to humans, and comprehensibility in terms of the associated conscious experience. Future work should seek to dissect the pharmacology of observed effects; while the psychedelic subjective effect in humans is strongly linked to the 5-HT_2A_ receptor, psilocin possesses a broad affinity for serotonin receptors. The significance of this is poorly understood and should not be neglected in studies of mechanistic underpinning, especially with regard to sleep regulation. Furthermore, understanding the time scale over which psilocybin-associated plasticity unfolds (and the corresponding effects on Process S) will be essential. While it is often assumed that plasticity must be induced during the acute experience, this is not necessarily guaranteed to be the case. If plasticity unfolds gradually over many days, or even selectively during sleep, this will not lead to changes visible in the EEG slow-wave activity.

We acknowledge as a limitation of this study that the head twitch response was not studied in depth. Head twitches were not clearly visible in the EMG among the high background activity and the video was not synchronised with EEG with sufficient precision to explore its EEG correlates. The meaning of the head twitch response remains unclear and revealing more of its neural mechanism could shed light on its value as a psychedelic state marker in rodents. However, it should be noted that the observed head twitch response in this study was not especially striking and occurred alongside a broader pattern of motor changes suggestive of restlessness. Head twitches were more common while the animal was active and ambulating and our observations do not support the possibility that head twitches themselves are the major source of sleep disruption.

From the clinical perspective, sleep represents an overlooked aspect of physiology in the efforts to understand how psychedelic-mediated mechanisms yield psychological benefits. The greatest challenge will be to draw mechanistic links between animals and humans to determine whether the physiology of psychedelic action translates. This is particularly important since therapeutic benefits in humans are dependent on appropriate psychological support, such as from a trained therapist. Identifying common mechanisms of action of psychedelics in both humans and rodents would be a great advance, and will require careful use of comparator drugs, with known pharmacological profiles and subjective effects, alongside translational tools such as EEG. Although many essential questions remain, a consistent picture of psychedelic biology is gradually forming, carrying the great potential to inform neuroscience in areas spanning basic neurobiology to clinical practice.

## Supplementary information

Suppl. Video 1

## Data Availability

The Matlab code scripts used to perform analyses and statistics are available on request.
